# Supporting school teachers’ rapid engagement with online education

**DOI:** 10.1007/s11423-020-09839-5

**Published:** 2020-10-08

**Authors:** Serdar Abaci, Judy Robertson, Holly Linklater, Fiona McNeill

**Affiliations:** 1 Moray House School of Education and Sport, University of Edinburgh, Edinburgh, EH8 8AQ UK; 2 School of Informatics, University of Edinburgh, Informatics Forum, Edinburgh, EH8 9AB UK

**Keywords:** Teacher education, Professional development, Online and blended learning

## Abstract

In response to Philipsen et al.'s (Educ Technol Res Dev 67:1145–1174, 2019) article titled “Improving teacher professional development [TPD] for online and blended learning [OBL]: a systematic meta-aggregative review”, we apply their proposed framework of important components of TPD for OBL to the support we provided to primary and secondary teachers as they engaged with online education during the COVID-19 pandemic. We reflect on observations of particular challenges for school teachers and the reasons behind them. While this framework is a useful reflection tool to guide professional learning for teachers beyond the emergency situations, we found that it is biased towards TPD for OBL in higher education settings. Thus, we suggest future work to differentiate educational levels in order to account for operational differences.

## Introduction

The COVID-19 pandemic created an unprecedented need for quick support and professional development for teachers across the globe to switch from in-class, face-to-face teaching to online teaching. Hodges et al. (2020) define teaching online without much planning, training, or preparation as “emergency remote teaching (ERT)” to differentiate it from planned, high-quality online teaching. In response to this crisis, a university-based team whose work focuses on data and digital education for schools offered weekly online webinars and a week-long online conference to support teachers in primary and secondary education from April to June 2020. These events were well attended (approximately 900 unique participants over a series of eight seminars and about 550 participants for the online conference). They included talks by academics and experts in teacher education and digital/data education, as well as teachers/head teachers who shared their experiences.

**Fig. 1:**
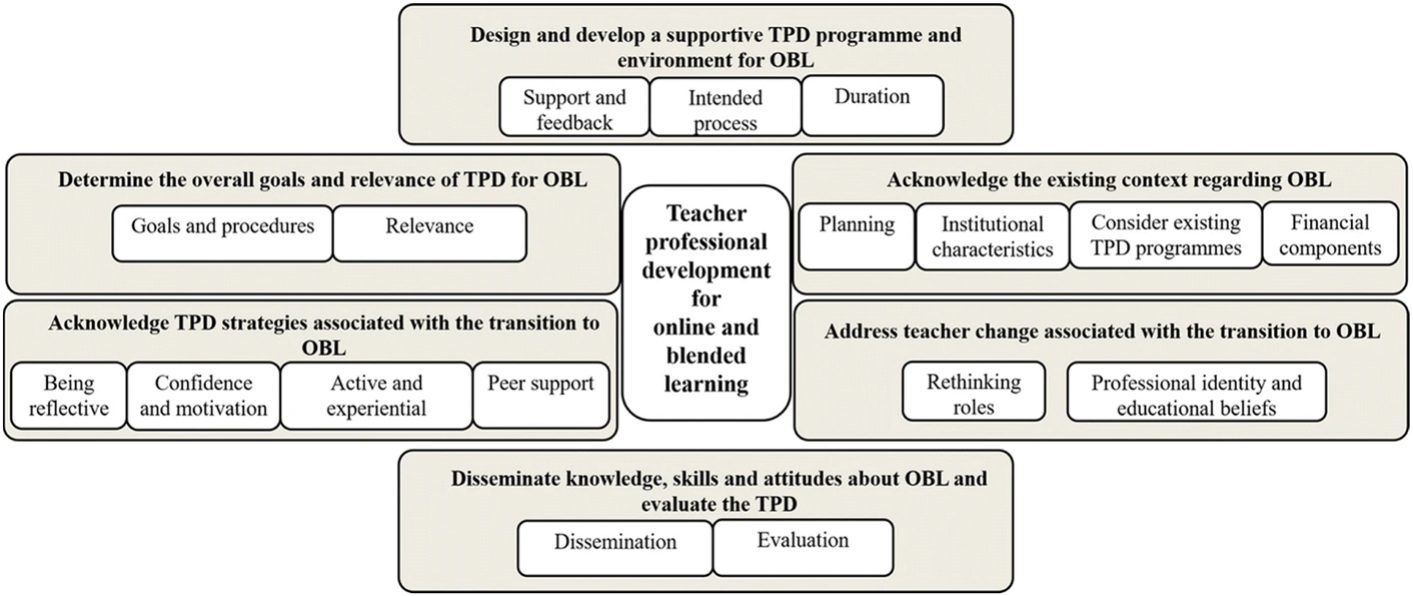
Comprehensive framework of important components of TPD that targets OBL. Reprinted with permission from Philipsen et al. (2019)

Philipsen et al.’s article presents a comprehensive and actionable framework of components of teacher professional development (TPD) for online and blended learning (OBL), based on a well-defined theoretical model (Desimone 2009) and “unequivocal and credible” qualitative data from a review of 15 published studies (Philipsen et al. 2019, p. 1149). It is important to note, however, that all but one of the reviewed studies were situated in a higher education context. This framework is a result of their systematic review and is composed of six synthesized findings. Each synthesized finding (SF) leads to an action recommendation. These are presented as “important components” in the framework model (Fig. 1). In this response we apply the proposed framework to the ERT support we provided to primary and secondary teachers, reflecting on observations of particular challenges for school teachers and the reasons behind them. Our responses align with the synthesized findings (SF).

## SF1: design and develop a supportive TPD programme and environment for OBL

From the outset of the ERT, teachers were in the position of creating a set of teaching solutions depending on how their school, local education authority, and government education agency provided resources and information. Universities and technology providers, as well as informal professional networks (e.g., Twitter) contributed to these processes. As a result, learning was opportunistic rather than planned and there was no clear duration due to the uncertainty about when schools would return to a normal, pre-COVID-19 state. Conversations and collaborations with colleagues in similar positions became critical to professional development. Based on both the invited contributors to our webinars and the participants, we suggest that collaboration, reflection, and evaluation of successes and limitations of online teaching are key considerations while designing TPD for OBL. In addition, support for pupils and their families was critically important throughout the lockdown period of schooling.

## SF2: acknowledge the existing context regarding OBL

We observed that those schools and education authorities who had previously invested in technology and professional development for digital learning, had a greater capacity to respond quickly towards online communication and teaching. On the other hand, teachers who had not previously used virtual communication platforms and who felt constrained by the availability of technology resources faced a steep learning curve. Another contextual element is that the engagement and support of parents and care givers was critical for successful online learning (Borup et al. 2013; McCarthy and Wolfe 2020). Support and guidance for schools—state and independent—was variable across Scotland, and previous national strategic guidance for professional learning and school improvement (e.g., Scottish Government 2016, 2019) were insufficiently nuanced to be constructive in the climate of a public health emergency.

## SF3: address teacher change associated with the transition to OBL

The pandemic required a rapid but radical shift of thinking about the role of a teacher. Professional identities within the Scottish education system are often constructed around the value that face-to-face personal contact is of primary importance. This is coupled with a cultural concern that too much screen time is “bad” for children in terms of distraction from studies, or detrimental to physical or mental health. During the pandemic, teachers were in a position of educating children entirely on screens. At the time of writing (September 2020), primary and secondary schools in Scotland are open full-time. For some teachers, this may have offered a sense of relief that they returned to being “proper” teachers. Other teachers were keen not to forget the lessons they learned from this potentially brief interruption to their professional learning and wanted to explore how digital learning practices they developed during the pandemic could transfer to “normal” practice.

In our view, educational policy makers, initial teacher education institutions, and the teacher professional bodies should develop a clear vision of what it means to be a teacher in a world which, shaken by a pandemic, includes the capacity to practice outside physical classrooms. This should include technical skills, pedagogical expertise, and judgement about online and blended learning. For resilience to further disruption from coronavirus or future crises, the capacity to calmly adapt teaching to incorporate digital and blended learning where necessary should be part of a teacher’s professional identity.

## SF4: determine the overall goals and relevance of TPD for OBL

As experienced teacher educators, we were struck by the change in teacher attitudes to professional learning about OBL. Previously, it had been difficult to persuade teachers to prioritise their time for TPD about digital learning; during the pandemic it was immediately relevant and highly sought after. Where online learning might previously have been associated with a “removed” or distant model of pedagogy, it now became the means for communities to stay connected and in this way meet teachers’ deep need to communicate with and care for the students and their families.

## SF5: acknowledge TPD strategies associated with the transition to OBL

We structured the online seminars to offer a mixture of peer support through presentations by fellow teachers and theoretical ideas which would assist the teachers in the audience to reflect on their own emerging practice. As the weeks progressed, we found the teachers sharing stories of their practice, encouraging and motivating each other through the text chat feature of the online conferencing tool. While active and experiential learning was not feasible in our setting, the seminars offered another channel for reflection and peer-support.

## SF6: disseminate knowledge, skills, and attitudes about OBL and evaluate the TPD

Teachers participating in our webinars also used the text chat facility to exchange examples and experiences of practice. We trusted that participating teachers and administrators would disseminate newly gained ideas and skills to colleagues within their schools. Evaluation of our webinar series to offer an additional set of guidance and perspectives on OBL for teachers, students, and parents, will be undertaken as part of a broader research inquiry into the changing landscape of teachers engagement in opportunities for professional learning relevant to digital and data education.

## Conclusion

Philipsen et al. (2019) provide a valuable framework for reflection as we start to think about how we can develop professional learning for teachers after the immediate emergency situation. The pandemic has resulted in conditions for TPD for OBL which are far from those recommended in the paper. Emergency remote teaching required an important skill set for teachers and student teachers alike, which has relevance to non-emergency provision and practice. A commitment to systematic professional learning in relation to the pedagogical skills relevant to online teaching is required and this framework could be a helpful way to guide the development of such a programme (or programmes). The components identified as “being reflective” and “active and experiential learning” are especially helpful to encourage course developers to focus on pedagogy and teacher learning, rather than solely delivering “teacher training” on narrow technical topics.

Application of the proposed framework to our context has also shown that the framework is biased towards TPD for OBL in higher education settings due to the context of studies reviewed in the paper. Schools and higher education institutions operate within different parameters such as institutional autonomy, in-house resources and staff for professional development, and student engagement and parental involvement in online learning. Therefore, we suggest that future versions of the framework should differentiate between these settings.
